# Intermolecular Forces: From Atoms and Molecules to Nanostructures

**DOI:** 10.3390/molecules27103072

**Published:** 2022-05-11

**Authors:** Jorge M. C. Marques, Frederico V. Prudente, Fernando Pirani

**Affiliations:** 1 CQC-IMS, Department of Chemistry, University of Coimbra, 3004-535 Coimbra, Portugal; 2 Instituto de Física, Universidade Federal da Bahia, Salvador 40170-115, Brazil; 3 Dipartimento di Chimica, Biologia e Biotecnologie, Università degli Studi di Perugia, Via Elce di Sotto 8, 06123 Perugia, Italy; 4 Dipartimento di Ingegneria Civile ed Ambientale, Università degli Studi di Perugia, Via G. Duranti 93, 06125 Perugia, Italy

Intermolecular forces, determined by the critical balance of interacting components having physical and chemical natures, control most of the static and dynamic properties of matter such as their existence in solid, liquid and gaseous phases, with their relative stability, and their chemical reactivity. In particular, they form simple interacting systems that represent the weakly bound precursor states of myriad phenomena. In some cases, such states promote the following nucleation, leading to the formation of large clusters and nanostructures, and in others, they open the passage to specific configurations of the reaction transition state of elementary reactions. Therefore, the characterization of intermolecular forces at a high level of detail is a question of crucial interest in several advanced research fields and represents a challenge for both experimentalists and theoreticians that will open new avenues in the study of systems of increasing complexity and of applied interest.

The core of this Special Issue is on the building up process that controls the transition from simple no covalent adducts to complex chemical structures. This Research Topic aims to underline the relationship between forces established among the particles of the system and other properties across different scales, i.e., from molecules, simple aggregates or small clusters to nanostructures and other types of matter at the mesoscale.

The Special Issue has 13 papers written by 50 authors from different countries with theoretical or experimental contributions (or both) in this research area. The work of González-Veloso et al. [1] unravels the interactions in magnetic ionic liquids (MILs) by applying Symmetry-Adapted Perturbation Theory (SAPT) for two different cations and several metal halide anions. The main contribution to the interaction energy is the electrostatic component, followed by the dispersion one in most of the cases. Furthermore, the calculated SAPT interaction energies had a good correlation with the experimentally measured melting points for these MILs, suggesting that the SAPT interaction energy could be used to predict melting points.

The paper from Espinosa-Garcia and co-workers [2] presents the first analytical full-dimensional Potential Energy Surface (PES) for the hydrogen abstraction CN + NH_3_ reaction, which is based on high-level ab initio calculations. The authors performed rigorous tests to analyze the quality and accuracy of the PES, from the Quasi-Classical Trajectory (QCT) calculations, and the results were compared with available experimental data. In turn, Raczyński et al. [3] have investigated the competition of intra- and intermolecular forces in anthraquinone and in some of its derivatives. These authors have employed Møller–Plesset second-order perturbation theory (MP2), Density Functional Theory (DFT) and time-dependent DFT (TD-DFT) methods to study the properties of intramolecular hydrogen bonds: the bridged protons are on the donor side for the electronic ground state, while secondary minima were found at the acceptor side in two excited states, which opens the possibility for excited-state intramolecular proton transfer (ESIPT). Concerning the intermolecular contacts, they are favored by the presence of nitro substituents.

The role of σ-hole in non-covalent interactions has been analyzed in complexes of ArBeO with simple ligands (*L*) by Borocci et al. [4]. These authors employed methods of bonding analysis to discern the factors associated with the transition from the essentially dispersive domain of *L*Ar to the σ-hole domain of the *L*-ArBeO complexes. In another methodological contribution, Vázquez and collaborators [5] have proposed improved corrections for amides and amines within the framework of the recently proposed PM6-FGC semi-empirical quantum mechanical approach. Substantial improvements in the calculation of noncovalent interactions are achieved when methylamine and acetamide are used as representatives of amine and amide groups.

The importance of the solvent effect in nonpolar solvents for the pyridone–hydroxy–pyridine tautomeric equilibrium and the dissociation of alkali metal halides have been studied by Shenderovich and Denisov [6]. Specifically, they analyzed how the modeling results change in connection with the polarizable continuum model (PCM) and with the use of the Adduct under Field (AuF) approach to nonpolar solvents, and how the AuF approach can simulate the dissociation of alkali metal halides. The solvation of Ca^+^ with helium has been investigated at the atomic level by Bartolomei et al. [7] in a combined experimental and theoretical work, which employs quantum Monte Carlo calculations and basin-hopping structure optimization based on the improved Lennard–Jones (ILJ) pair potential, while a reflectron time-of-flight mass spectrometer was applied to analyze the product ions ejected. These authors concluded that 25 helium atoms complete the first solvation shell (the optimum packing of He*_N_*Ca^+^ is N≈17), and the corresponding clusters are essentially liquid-like structures. Oliveira et al. [8] have also employed the ILJ function to model the complete family of rare-gas diatomic molecules by fitting CCSD(T)/CBS energies. This work shows that most of the LJ inadequacies both at large and short intermolecular distances are corrected by ILJ. Indeed, the rovibrational spectroscopic constants calculated by the ILJ function show a more effective agreement with experiment than those obtained with the LJ potential model.

The importance of intermolecular forces in the ionic polymerization process on large clusters of acetylene molecules to form polyaromatic hydrocarbons (PAHs) has been studied by Molina and Stein [9]. They employed ab initio molecular dynamics (AIMD) simulations within interstellar medium (ISM) conditions and showed that four acetylene units can aggregate to form C_8_$H_{8}^{+}$-bonded species, some of which are bicyclic structures. As for adsorption–desorption processes, the potential barriers resulting from a balance among the intervening intermolecular forces affect the corresponding low-pressure hysteresis in open slit-like micropores. Based on two thermodynamic models for the hysteresis loop, this phenomenon has been analyzed within the framework of thermodynamically irreversible processes and fractal geometry by Dragan et al. [10].

The formation of complex supramolecular structures via self-assembly of small molecules is a phenomenon also conducted by intermolecular forces. An example is the competition between hydrogen bonding and aromatic-rings stacking, which may lead to a dramatic change in the structure of biological systems. By using supersonic jet expansions, Zimmermann et al. [11] have prepared mixture of phenol, acetophenone and six of its halogenated derivatives and detected the formation of complexes through infrared spectroscopy, which allowed to observe that halogenation has a great effect on the docking site, with a wide range of energies for either the phenyl or the methyl side of the ketone. In the phenol complex with nonhalogenated acetophenone, by contrast, methyl docking is the most prevailing one.

Fernández et al. [12] have applied DFT calculations to analyzed the supramolecular helical aggregation of oligo(phenyleneethynylene) monomers. From the minimum structures obtained at the DFT/6-31G** with the dispersion-corrected B3LYP-D3 functional, these authors calculated absorption and circular dichroism spectra at the TD-DFT (CAM-B3LYP/3-21G) level and concluded that the theoretical results display good agreement in comparison with available experimental spectra. Finally, Prudente and Marques [13] have characterized the thermodynamic signatures of both structural transitions and dissociation of charged colloidal clusters that are modeled with pair-potentials comprising a short-range attractive contribution and a long-range repulsive component. The competition between attractive and repulsive interactions may lead to the formation of metastable structures with spherical-like geometries (for small clusters), Bernal spirals (for intermediate-size clusters) and beaded-necklace shapes (for large-size clusters) that can survive at non-zero temperatures.

Thus, as we have seen, these papers covered different aspects of the research presently carried out on intermolecular forces and showcase the diversity of methodologies employed by theoreticians and experimentalists, making this Special Issue an interesting contribution to this field of knowledge.

## Data Availability

Data are contained within the article.
